# Genetic variation for effects of drought stress on yield formation traits among commercial soybean [*Glycine max* (L.) Merr.] cultivars adapted to Ontario, Canada

**DOI:** 10.3389/fpls.2022.1020944

**Published:** 2022-10-13

**Authors:** Michael Gebretsadik Gebre, Istvan Rajcan, Hugh James Earl

**Affiliations:** Department of Plant Agriculture, University of Guelph, Guelph, ON, Canada

**Keywords:** Drought stress, genetic variation, soil water deficits, soil water extraction, soybean, water use efficiency, yield components, seed yield ratio

## Abstract

Drought stress significantly limits soybean [*Glycine max* (L.) Merr.] yields in Ontario, Canada. Many studies of genetic variation for drought tolerance compare commercial lines with exotic, unadapted germplasm. We hypothesized that even current commercial cultivars adapted to Ontario would differ significantly for traits related to drought tolerance. In a greenhouse experiment, we grew fifteen soybean cultivars in field soil amended with sand in 1-m rooting columns, which allowed for simulation of field-like soil water profiles and rooting depths. Two watering treatments were imposed from the first flower until maturity by daily restoration of soil water to either 100% (control), or 50% (drought stress) of the maximum soil water holding capacity. Throughout the experiment, we measured volumetric soil water content at different depths in the soil profile, but found no evidence at any developmental stage that the cultivars differed for their ability to extract soil water from different depths. Drought stress reduced seed yield by 51% on average. Similar to the effects of drought in the field, pod number was the yield component most affected, with effects on seeds per pod and single-seed weight being comparatively minor. There were significant cultivar **×** treatment interactions for seed yield, pod number, shoot dry matter, and water use. We identified two drought-sensitive (*Saska* and *OAC Drayton*) and three drought-tolerant (*OAC Lakeview*, *OAC Champion*, and *PRO 2715R*) cultivars based on their ratios of seed yield under drought stress to seed yield under control conditions (seed yield ratio, SYR). Regression and principal component analyses revealed that drought-tolerant (high-SYR) cultivars were consistently those that maintained relatively high values for water use, biomass accumulation and pod number under drought stress; high water use efficiency under drought stress was also associated with a high SYR. One of the cultivars, *OAC Lakeview*, displayed a distinct mode of drought tolerance, maintaining a very high fraction of its control pod number under drought stress. This study helps define the physiological basis of soybean cultivar differences in drought tolerance, and provides direction for soybean breeders to select traits that could improve yield under drought stress.

## Introduction

Soybean [*Glycine max* (L.) Merr.] is the most valuable oilseed crop grown in Ontario, Canada, where it is typically produced under rainfed conditions. Drought stress occurring during the critical stages of the crop’s development can significantly reduce its yield potential (Hufstetler et al., 2007; Visser, 2014). Recent research demonstrates that drought stress constitutes a significant constraint to Ontario’s soybean yield in most growing seasons. Reported yield losses attributed to drought stress in field experiments ranged from 8 to 24% and supplemental irrigation during the reproductive stage under field conditions was observed to significantly increase soybean yield, largely by increasing the number of pods per plant (Earl, 2012; Visser, 2014). The occurrence of transient soil water deficits in the region especially during drier years may lead to yield losses even exceeding 25% (H. J. Earl, unpublished data). Drought stress in Ontario soybean mainly occurs during the critical periods of pod-setting and seed-filling, typically from July to August when the crop’s growth rate and daily water use are highest. Such yield-limiting soil water deficits frequently occur with no obvious outward signs of water stress such as leaf wilting, but result in fewer pods, reduced single-seed weight, and hastened crop maturity, which shortens the seed-filling period and finally reduces seed yield (Snyder et al., 1982; Brevedan and Egli, 2003; Visser, 2014; Giordani et al., 2019).

In this scenario of mild but cumulative soil water deficits in Ontario soybean production, targeted improvement for drought stress tolerance among adapted commercial cultivars is vital. Identification of physiological traits underlying tolerance to drought stress for specific regions would benefit from this effort. To do so, traits need to be measured accurately in controlled environments to pinpoint specific processes that may be missed in field trials. Previous studies suggest that there is a benefit to the adoption of controlled environment phenotyping methods that permit the use of mineral soils and produce field-like soil water profiles and root biomass distributions by depth (Gebre, 2020; Gebre and Earl, 2020; Gebre and Earl, 2021).

It has been reported that commercial soybean cultivars have a wide genetic variation for physiological traits related to drought stress tolerance such as (a) whole-plant water use efficiency (WUE), (b) regulation of whole-plant water use in response to soil water content, and (c) leaf epidermal conductance to water vapor when stomata are closed, also known as dark-adapted leaf conductance which predicts WUE of soybean (Walden-Coleman et al., 2013; Visser, 2014). The genetic variation in soybean for the critical soil water content at which water use begins to decline (Hufstetler et al., 2007) indicates that different genotypes make differing “decisions” about how to respond to reduced water availability. Moreover, genetic variation for WUE among Ontario soybean cultivars has been reported to significantly impact the cultivars’ relative susceptibilities to yield loss under naturally-occurring drought stress conditions in the region (Visser, 2014).

Oya et al. (2004) reported a wide genetic variation for drought tolerance (~25 to 44%) among ten Brazilian soybean cultivars tested across two growing seasons. The authors concluded that higher drought tolerance in those soybean cultivars, which they defined to be higher yielding under drought stress, was correlated with maintaining a higher crop growth rate under drought stress conditions, especially during the early reproductive developmental stages. They also speculated that a higher vegetative growth together with the capacity to remobilize photo-assimilates from the vegetative organs (leaves, stems, and pericarps) to the reproductive sinks (e.g., seeds; supporting the seed growth rate) under drought stress could be considered as physiologically important traits contributing to soybean drought tolerance.

Drought stress affects soybean yield through a reduction of any of its yield components (pod number, seeds per pod, single-seed weight) (e.g., Gebre, 2020; Gebre and Earl, 2021). Moreover, yield loss in soybean can result from soil water deficits occurring at any time, though the magnitude of the yield reduction depends on the stage of development, the timing of the stress, and the severity of the stress (Snyder et al., 1982). Brown et al. (1985) identified a soybean cultivar *‘Sohoma’* that displayed a significantly lower reduction in its seed yield and yield components (100-seed weight and seed number) under soil water deficit conditions in the field, as compared to three other cultivars. In that work, the authors imposed the drought stress treatments at either the R2 (full flowering) or R4 (full pod) developmental stage; stress imposed at R2 resulted in a greater reduction (~31%) in seed yield than stress imposed at R4 (~21%).

Many studies of genetic variation for drought stress tolerance and related traits compare elite commercial lines with exotic, unadapted germplasm (e.g., Oya et al., 2004; Hufstetler et al., 2007; Walden-Coleman et al., 2013; He et al., 2016; He et al., 2017; Fried et al., 2019; Giordani et al., 2019). We hypothesized that even current commercial soybean cultivars grown in Ontario would differ significantly for their response to drought stress in terms of their growth, yield and yield components. The specific objectives of the present study were to: (1) assess the effect of drought stress on seed yield, yield components (pod number, seeds per pod, single-seed weight), biomass, and water use for fifteen commercial soybean cultivars adapted to Ontario; (2) evaluate the genetic variation for yield formation and related traits contributing to drought tolerance, and the relationships among these traits; and (3) identify drought-sensitive and drought-tolerant cultivars based on their ratio of seed yield under drought stress: seed yield under control conditions (SYR).

## Materials and methods

### Plant material and culture system

**Figure 1:**
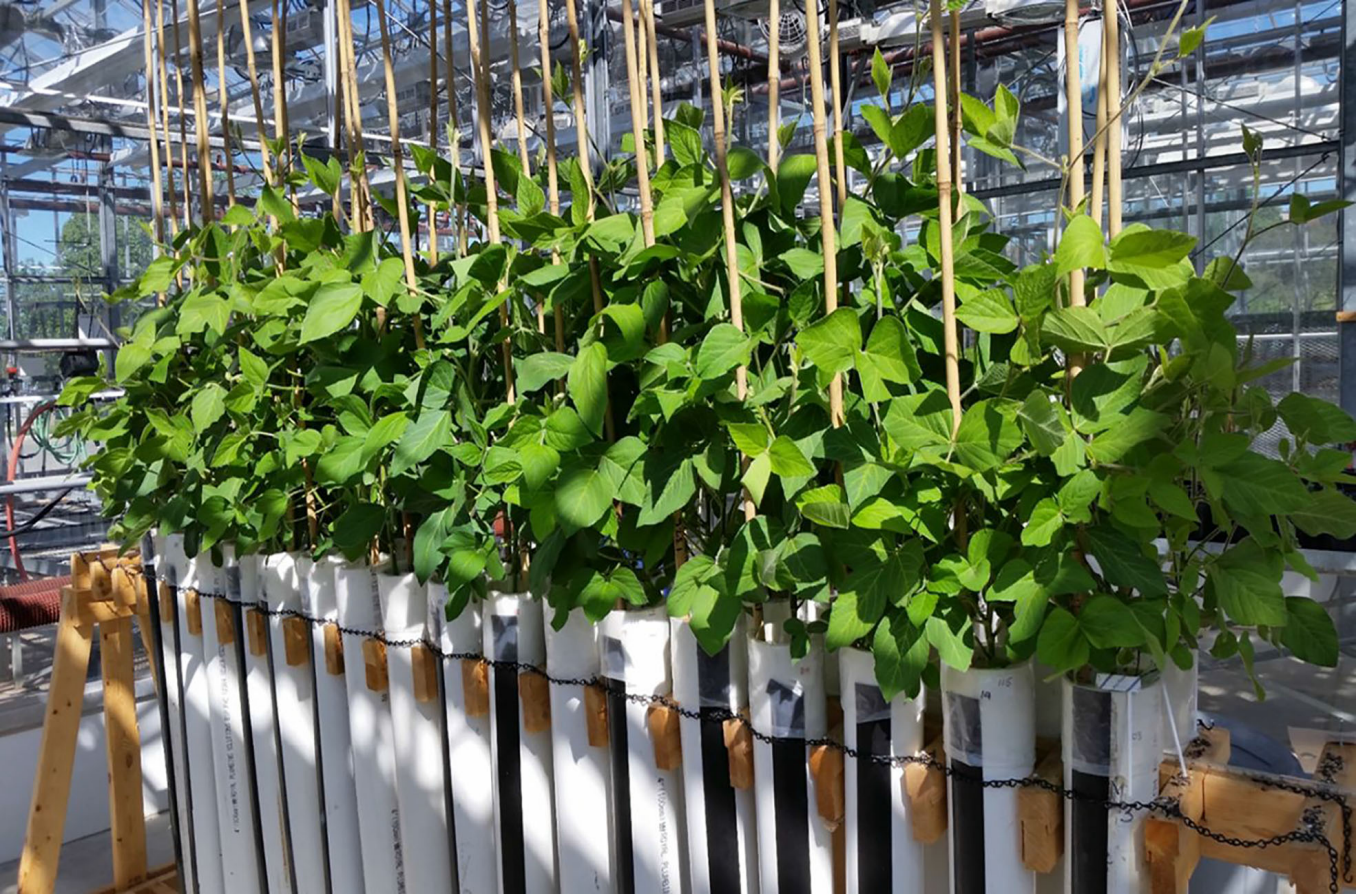
A single replicate of the study. The 15 soybean cultivars were grown in a greenhouse in 1-m PVC rooting columns (tubes). Tubes are drilled on the sides to allow for time-domain reflectometry measurements of volumetric soil water content. Plastic liners allow for the removal of intact root systems. The photo was taken at the R1 developmental stage.

Plants were grown in the Crop Science Building’s greenhouses at the University of Guelph (43.5314° N, -80.2244° W), Guelph, ON, Canada, in 2017 and 2018 using a culture system and drought stress simulation method developed and described in detail by Gebre and Earl (2020; 2021). Fifteen Ontario-adapted commercial soybean cultivars of similar maturity were selected based on days to maturity (DTM) ratings in the Ontario Oil and Protein Seed Crop Committee soybean cultivar trials brochure. These cultivars were from ten different public- and private-sector organizations: University of Guelph Ridgetown, Agriculture and Agri-Food Canada Ottawa, Mycogen, SeCan, Woodrill Farms, Prograin, La Coop Federee, Hendrick Seeds, Hensall District Coop, PRO Seeds, Bramhill Seeds, and Syngenta (**Table 1** **;** **Figure 1**).

**Table 1:** The 15 Ontario-adapted commercial soybean cultivars used in the greenhouse screening study.

Cultivar’s name	Supplier	Days to maturity
5A090RR2	Mycogen	117
Absolute RR	SeCan	120
Blade RR	Woodrill	117
Bruce	Prograin	115
Dares	La Coop Federee	113
DH420	Hendrick Seeds	117
HDC 2701	Hensall District Coop	114
OAC Champion	PRO Seeds	115
OAC Drayton	Bramhill Seeds	116
OAC Lakeview	SeCan	112
OAC Purdy	SeCan	118
PRO 2715R	PRO Seeds	120
S08-C3	Syngenta	116
Saska	Prograin	116
Wildfire	Woodrill Farms	117
Mean		116
S.E.		0.5

Days to maturity data represent the mean of two years (2011 and 2013) measured in a field experiment under rainfed conditions (Visser, 2014).

The plants were grown in 1 m long 10 cm diameter PVC rooting columns (tubes) lined with polyethylene liners, and PVC end caps at the bottom, each with a drainage hole. The soil mixture was a blend of six parts by volume field soil, two parts granitic sand (B-sand; Hutcheson Sand and Gravel Ltd., Huntsville, ON, Canada), and one part peat-based potting mix (PGX; Premier Tech, Brantford, ON, Canada).

The field soil used, classified as a *London loam* (Grey-brown Podzolic loam till), was collected from the topsoil (upper 15 cm) at the Elora Research Station (Elora, ON, Canada; 44.6837° N, -80.4305° W) that had a prior history of soybean production. It was a silty loam (silt **=** 50%, sand **=** 31%, clay **=** 19%, mineral components by mass) texture that contained 4.2% organic matter, 23.5 ppm P, 61.5 ppm K, 280 ppm Mg, 2375 ppm Ca, 15 ppm Na, 14.4 meq per 100 g CEC, and had a pH of 7.4 according to a soil test performed by A&L Laboratories Inc., London, ON, Canada. During the process of potting, the tubes were filled in a systematic fashion of loading and packing until the soil reached approximately 1 cm below the top of the tube. The total weight of each tube with its soil was then recorded. A commercial 20-20-20 N-P-K plus micronutrients fertilizer (Master Plant Products Inc., Brampton, ON, Canada) at the rate of 0.8 g tube^-1^ dissolved in 500 mL of water was added at the top of the soil surface in each tube so that the first 0-30 cm of the soil profile could be thoroughly wetted with the fertilizer solution.

**Figure 2:**
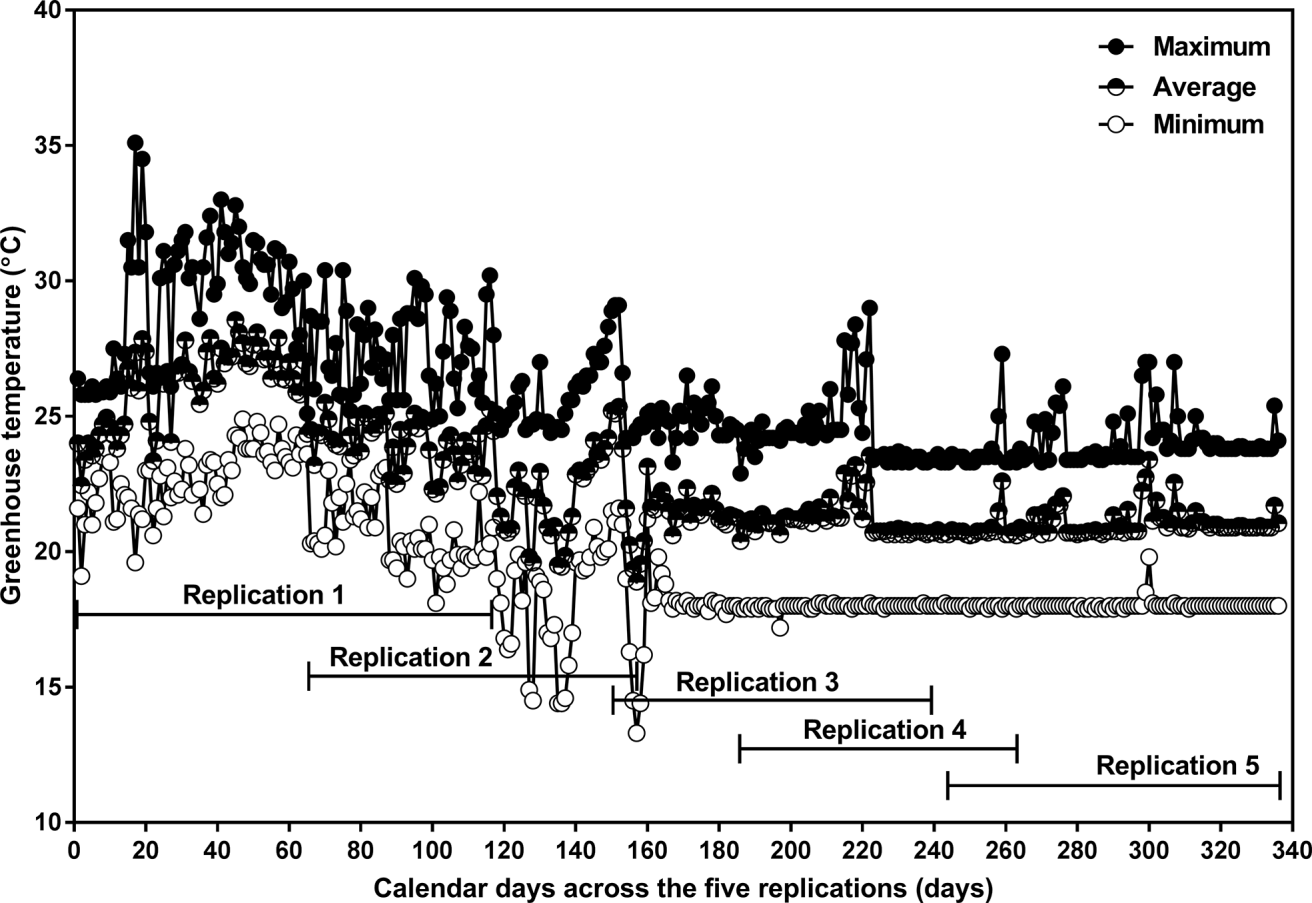
Daily minimum (open circle), average (half-closed circle), and maximum (closed circle) greenhouse air temperature as a function of calendar days from planting to physiological maturity in the 2017 summer and fall seasons, and the 2018 winter season. The experiment consisted of five sequential replicates. The planting dates for replications 1, 2, 3, 4, and 5 were April 29, July 2, September 25, October 30, and December 27 in 2017, respectively. The physiological maturity dates for replications 1, 2, and 3 were August 27 (122 days after planting; DAP), September 30 (90 DAP), December 22 (88 DAP) in 2017, and for replications 4, and 5 were January 15 (77 DAP) and March 29 (92 DAP) in 2018, respectively. Replication 1 was excluded from the data analysis due to extreme temperatures that occurred in the greenhouse.

The experiment was repeated sequentially, with sowing dates of April 29, July 02, September 25, October 30, and December 27, 2017, for the five replications. Four seeds were sown per tube at 3 cm depth, and then thinned after emergence to one seedling per tube. Target greenhouse temperatures were 25**°**C during the day and 20**°**C during the night with an average relative humidity of 80%. The actual greenhouse daily minimum, maximum, and average temperatures from planting to physiological maturity (R7 developmental stage; developmental staging as per Fehr et al., 1971 and Purcell et al., 2014) for each replication are provided in **Figure 2**. Natural sunlight was supplemented with overhead high-pressure sodium and metal halide lamps to provide a supplementary 400 µmol m^-2^ s^-1^ photosynthetic photon flux density at the top of the canopy during the photoperiod, and to provide daylength extension to achieve 16 h of light and 8 h of dark.

### Determining soil water holding capacity

To determine the soil water content and mass of dry soil in each tube, soil samples were taken during the potting process and dried in a forced-air drier at 80**°**C until a constant weight was attained. The maximum soil water holding capacity (SWHC) of the soil loaded in each tube was approximately 2.5 L (Gebre and Earl, 2020; Gebre and Earl, 2021). Thus, 24 h after wetting the first 0-30 cm of the soil profile with 500 mL of a fertilizer solution, about 3.5 L of water was added to exceed the maximum SWHC (against free drainage) of each tube. After another 24 h, the tubes were watered (~500 mL tube^-1^) again to free drainage to ensure maximum SWHC was achieved. Elastic bands were used to close the plastic liners at the tops of the tubes to prevent surface evaporation. The tubes were allowed to drain until a constant weight was achieved (tube weight at maximum SWHC). The weights of the dry soil and the tubes were then subtracted from this weight to determine the soil water content at maximum SWHC. Then, the target weight for each tube was calculated as the tube **+** soil dry weight, plus the water weight at maximum SWHC measured for that tube multiplied by the target fraction of the maximum SWHC (either 100 or 50%, depending on the watering treatment).

### Experimental design, treatments, and measurements

Each replicate of the experiment was arranged as a 15 **×** 2 split-plot design (15 **×** 2 **=** 30 tubes), with the 15 soybean cultivars as the main-plot factor and the two watering treatments (watered daily to either 100 or 50% of the maximum SWHC) as the sub-plot factor. The experiment consisted of five sequential replicates (replicated in time). The 30 experimental units (tubes) in each replicate were placed on a wooden stand (**Figure 1**), arranged in two rows of 15 tubes. Four tubes (two at each end) were used to grow border plants to minimize border effects.

Until the R1 (beginning flowering) developmental stage, all tubes were weighed and watered daily to their maximum SWHC. When more than 50% of the plants in a replicate had reached R1, watering treatments were imposed and lasted through the R8 (full maturity) developmental stage. During this period (R1 to R8 stages), tubes were returned to either 100% (control) or 50% (drought stress) of the maximum SWHC by daily weighing and watering. There was no leaching of water from the drainage holes in the bottoms of the tubes at any time during the experiment, so total plant water use (WU) per day was calculated from the water additions at the top of the tubes. The whole-plant WU from planting to harvest was calculated as WU (g plant^-1^) **=** [total amount of water added to each tube from planting to harvest **+** (starting weight **–** end weight of each tube at harvest) **+** whole-plant fresh biomass at harvest].

Time-domain reflectometry (TDR; Field Scout™ TDR 100 Soil Moisture Meter, Spectrum Technologies, Inc., Aurora, IL, United States) millisecond readings were recorded once per week for the duration of the study, from the planting date until the R7 (physiological maturity) stage. The TDR measurements were performed at five equally spaced points (at 10, 30, 50, 70, and 90 cm below the soil surface) *via* pre-drilled TDR access holes in the sides of the tubes. The TDR measurements were always made just before daily watering (i.e., 24 h after the previous watering). The volumetric soil water content (VSWC; %) was calculated from the TDR millisecond readings using a calibration curve developed for the specific soil mix used in this experiment (Gebre and Earl, 2020).

### Harvest and postharvest procedures

About 5 to 10 days after the full maturity date when 95% of the pods were brown (at the R8 developmental stage), all plants were cut at soil level and total aboveground plant fresh biomass was recorded. Immediately after harvest, the number of filled pods (pods with seeds) per plant (pod number; PN) was counted. All aboveground samples were then dried in a forced air drier at 80**°**C until a constant weight was attained. Then, the total aboveground (shoot) dry matter (SDM) per plant was determined, pods were threshed by hand, seed yield (SY, the weight of seeds per plant; g plant^-1^) was recorded, and the total number of seeds per plant was counted. Seeds per pod (SPP) was then calculated as the number of seeds divided by PN. Individual seed weight (SW; g seed^-1^) was calculated by dividing the SY by the seed number. Harvest index (HI), the fraction of SDM allocated to the seed, was calculated as the SY divided by the SDM for each plant. After harvesting the aboveground plant parts, the soil and the intact root systems within each rooting column were carefully removed by pulling out the plastic liner after laying the tube down on its side. The root samples were washed, placed into labelled paper bags, and then oven-dried in a forced air drier at 80**°**C until a constant weight was attained (typically 4 days) and then the final root dry matter (RDM) of each sample was recorded. Root-to-shoot ratio (R:S) was calculated as the ratio of RDM to SDM. Whole-plant water use efficiency (WUE; g L^-1^) was calculated by dividing total plant dry matter (TDM; RDM **+** SDM) by total WU from planting to full maturity.

### Statistical analyses

Analysis of variance was performed using the PROC GLIMMIX procedure of SAS Version 9.4 (SAS Institute Inc., Cary, NC, United States). A Type 1 error rate of 0.05 was used for all statistical tests. Since the dependent variables SY, PN, SPP, SW, SDM, RDM, R:S, TDM, HI, WU, WUE, and DTM were quantitative and continuous, a generalized linear mixed model (GLMM) was fitted with an identity link function and a Gaussian response distribution. The variances of the dependent variables were partitioned into the fixed effects of cultivar and watering treatments, and their interactions (cultivar **×** watering treatments), and the random effects of blocks, and block **×** cultivar interactions. We used the following statistical model:


$$Y_{ijk}=\;µ\;+\;c_{i}+\;w_{j}+\;c_{i}w_{j}+\;B_{k}+\;c_{i}B_{k}+\epsilon_{ijk}$$

where *Y_ijk_* denotes the value of the measured trait for the *i*th cultivar treatment (15 cultivars) of the *j*th watering treatment (control or drought stress) in the *k*th block, *µ* is the grand mean, *c_i_* is the cultivar treatment effect (i.e., the main-plot factor), *w_j_* is the watering treatment effect (i.e., the sub-plot factor), *c_i_w_j_* is the interaction effect between cultivar and watering treatments, *B_k_* is the effect of the *k*th block (treated as a random effect), *c_i_B_k_* is the main-plot random error (treated as a random effect), and *ϵ_ijk_* is the residual.

For each day on which VSWC was measured, the repeated measures analysis of variance of VSWC by depth was partitioned into the fixed effects of cultivars, watering treatments, and depth, and their interactions (cultivar **×** water, cultivar **×** depth, water **×** depth, and cultivar **×** water **×** depth), and the random effects of blocks, and block **×** cultivar interactions. The random interaction term *subject* ***×*** *depth* was included in the model where the *subjects* (tubes) were assumed independent (identity covariance structure) and for *depth* three possible types of covariance structures [*compound symmetric*, CS; autoregressive *order 1*, AR(1); and *heterogeneous autoregressive order 1*, ARH(1)] were compared. In each case, the most appropriate model was selected based on AICC and BIC fit statistics, no overdispersion based on the generalized Chi-square/df, and assessment of conditional studentized residual plots. Since the spacing interval between VSWC by depth measurements was equally spaced, the *Kenward-Roger* adjustment for bias correction for the denominator degrees of freedom was applied (Kenward and Roger, 1997).

*F*-tests and log-likelihood ratio tests were used to determine the significance of fixed and random effects, respectively. Least-square means comparisons were performed using a Protected Fisher’s LSD test. The assumptions for the GLMM, in particular, random and normally distributed experimental errors and constant (homogeneous) error variance were tested by (1) plotting the studentized residuals against factor levels and predicted values; (2) generating a Q-Q plot and scatterplots of the residuals versus fitted values; and (3) performing a formal test of normality using a Shapiro-Wilk. Putative outliers, if any, were detected if the values of the studentized residuals were not within the range of -3.4 to 3.4 (Bowley, 2015).

Drought tolerance of each cultivar was quantified as the drought stress seed yield ratio (SYR), defined as the fraction of a cultivar’s seed yield per plant under control conditions (100% SWHC) that was maintained under drought stress conditions (50% SWHC), i.e., SYR **=** SY per plant (drought stress)**/**SY per plant (control). The genetic variation for the various physiological traits was calculated using the cultivar least square means as the [(maximum value **-** minimum value)/minimum value **×** 100]). The relationships among selected yield formation and related traits contributing to drought tolerance were investigated *via* correlation and regression analyses using the PROC CORR and PROC REG procedures in SAS Version 9.4 (SAS Institute Inc., Cary, NC, United States). The biplot display of principal component analysis (PCA) was also used to determine the relationship between multiple traits (drought stress to control ratio values) using the Multivariate procedure of PAST Software Version 3.25 (Hammer et al., 2001).

## Results

Extreme temperatures occurred in the greenhouse during Replication 1 (**Figure 2**); data from this replicate were excluded, and so all analyses were based on the remaining four replicates.

### Effects of drought stress on soybean yield, yield components, and related traits

**Table 2** shows the main effects of drought stress treatment on soybean yield, yield components and other related traits. The drought stress treatment had a significant effect on every trait except HI. It reduced SY by 51% relative to the control (*p* < 0.0001), PN by 51% (*p* < 0.0001), SPP by 4% (*p* < 0.01), SDM by 51% (*p* < 0.0001), RDM by 36% (*p* < 0.0001), TDM by 48% (*p* < 0.0001), WU by 51% (*p* < 0.0001), and DTM by 4 days (*p* < 0.0001). The drought stress treatment increased single SW by 6% (*p* < 0.0001), R:S by 35% (*p* < 0.0001), and WUE by 6% (*p* < 0.0001), as compared to the control watering treatment (**Table 2**). Roots were extensively nodulated in both watering treatments.

**Table 2:** Effect of watering treatments [control (100% soil water holding capacity; SWHC), and drought stress (50% SWHC) on yield, yield components, and related traits.

Yield and related traits	Watering treatments					
	Control	Stress	Mean	Standard error	Difference (%)	*p* Water^‡^	
Seed yield (g plant^-1^)	24.3	12.0	18.1	1.17	-51	**<0.0001**	
Pods per plant	63.1	30.6	47.0	2.69	-51	**<0.0001**	
Seeds per pod	2.26	2.16	2.21	0.062	-4	**0.0017**	
Seed weight (g seed^-1^)	0.175	0.186	0.180	0.0073	6	**<0.0001**	
Shoot dry wt. (g plant^-1^)	48.6	23.8	36.2	2.48	-51	**<0.0001**	
Root dry wt. (g plant^-1^)	13.1	8.4	10.7	0.78	-36	**<0.0001**	
Root: shoot (g g^-1^)	0.27	0.37	0.32	0.033	35	**<0.0001**	
Total dry wt. (g plant^-1^)	61.7	32.2	47.0	2.81	-48	**<0.0001**	
Water use (L plant^-1^)	32.2	15.6	24.0	0.88	-51	**<0.0001**	
Total dry wt. WUE (g L^-1^)	1.92	2.04	1.98	0.072	6	**<0.0001**	
Harvest index (g g^-1^)	0.50	0.50	0.50	0.013	0.3	0.7251	
Days to maturity (days)	102	98	100	2.9	-4	**<0.0001**	

Each value is the mean of four replications averaged across the 15 cultivars tested. The percent difference between treatments is calculated as (Control – Stress)/Control **×** 100.

**^‡^** Significant watering treatment effects (*p* < 0.05) are indicated in bold.

### Effects of cultivar and drought stress on yield, yield components, and related traits

The cultivars differed significantly (*p* < 0.001) for every parameter measured. **Table 3** shows the cultivar by treatment interaction means for those traits for which there was a significant cultivar **×** treatment interaction effect: SY, PN, SDM, and WU. Traits for which there were significant cultivar effects but no interaction (SPP, SW, RDM, TDM, R:S, WUE, HI, and DTM) are presented in **Table 4**. The overall coefficient of variation (CV) for SY was low (9%) while the other CVs ranged from 8 to 13%.

**Table 3:** Cultivar and watering treatment interactive effects on seed yield (SY), pod number (PN), shoot dry matter (SDM), and water use (WU) for 15 soybean cultivars grown in a greenhouse in 1-m rooting columns under two watering treatments [control (100% soil water holding capacity; SWHC), and drought stress (50% SWHC)].

Yield and related traits	SY(g plant^-1^)		PN(pods plant^-1^)		SDM(g plant^-1^)		WU(L plant^-1^)	
Cultivar	Stress	Control	Stress	Control	Stress	Control	Stress	Control
5A090RR2	12.8	26.0	35.0	70.8	24.5	49.4	16.0	33.1
Absolute RR	12.5	26.1	31.8	68.3	23.5	50.0	15.2	31.3
Blade RR	11.8	24.8	32.5	75.8	22.9	46.6	15.5	31.4
Bruce	10.8	22.2	27.0	60.8	23.8	51.8	15.9	34.3
Dares	11.5	23.6	27.5	54.0	24.8	51.2	15.3	31.7
DH420	11.4	22.7	25.8	47.8	22.1	42.6	15.3	30.5
HDC 2701	10.8	23.4	21.5	51.8	21.4	48.3	15.0	30.7
OAC Champion	12.4	22.9	25.3	50.0	25.0	45.6	15.3	28.6
OAC Drayton	12.8	28.6	31.8	73.3	23.9	56.7	15.9	33.7
OAC Lakeview	14.7	24.5	37.8	58.0	25.5	45.4	15.8	28.5
OAC Purdy	12.0	25.4	30.5	66.5	24.3	48.6	16.1	34.2
PRO 2715R	12.3	22.1	37.3	77.0	28.9	54.8	16.9	33.7
S08-C3	11.4	24.0	35.5	64.8	22.0	45.0	14.7	30.8
Saska	11.9	27.0	35.5	76.8	22.8	51.0	15.4	37.0
Wildfire	10.7	20.9	24.3	50.8	21.7	42.4	16.2	33.7
S.E.	1.50		4.16		3.09		1.28	
CV (%)	9.45		13.25		9.71		8.28	
*p* Cultivar (C) **^‡^**	**0.0024**		**<0.0001**		**0.0010**		**0.0004**	
*p* Water (W) **^‡^**	**<0.0001**		**<0.0001**		**<0.0001**		**<0.0001**	
*p* C **×** W**^‡^**	**0.0212**		**0.0037**		**0.0317**		**0.0171**	

Each value is the mean of four plants, one from each of the four sequential replications. Data are presented only for those traits that showed a significant (*p* < 0.05) cultivar by water interaction effect.

**^‡^** Within a measured trait, significant cultivar, water, and cultivar by water interaction effects (*p* < 0.05) are indicated in bold.

**Table 4:** Cultivar effects on seeds per pod (SPP; seeds pod^-1^), single-seed weight (SW; g seed^-1^), root dry matter (RDM; g plant^-1^), total dry matter (TDM; g plant^-1^), root-to-shoot ratio (R:S; g g^-1^), harvest index (HI; g g^-1^), whole-plant DM-based water use efficiency (WUE; g L^-1^), and days to maturity (DTM; days) for 15 soybean cultivars grown in a greenhouse in 1-m rooting columns under two watering treatments [control (100% soil water holding capacity; SWHC), and drought stress (50% SWHC)].

Cultivar	SPP	SW	RDM	TDM	R:S	HI	WUE	DTM
5A090RR2	2.30	0.158	15.4	52.3	0.46	0.53	2.19	104
Absolute RR	2.24	0.175	10.8	47.5	0.31	0.53	2.04	103
Blade RR	2.35	0.146	14.0	48.8	0.43	0.52	2.08	106
Bruce	2.24	0.174	13.3	51.0	0.36	0.44	2.04	101
Dares	2.36	0.181	9.1	47.1	0.26	0.46	2.02	103
DH420	2.27	0.203	8.1	40.5	0.27	0.52	1.8	95
HDC 2701	2.16	0.220	9.9	44.7	0.33	0.50	1.95	96
OAC Champion	2.45	0.195	10.2	45.4	0.31	0.50	2.10	98
OAC Drayton	2.23	0.184	10.4	51.7	0.27	0.52	2.07	101
OAC Lakeview	2.09	0.195	9.1	44.5	0.27	0.56	2.02	99
OAC Purdy	2.05	0.191	10.3	46.7	0.30	0.51	1.89	94
PRO 2715R	2.07	0.149	12.6	54.4	0.33	0.42	2.19	107
S08-C3	2.02	0.173	9.5	43.0	0.29	0.53	1.88	98
Saska	2.21	0.158	8.0	44.9	0.24	0.53	1.76	99
Wildfire	2.08	0.205	10.5	42.5	0.34	0.50	1.73	103
Mean	2.21	0.180	10.7	47.0	0.32	0.50	1.98	100
S.E.	0.085	0.0094	1.19	3.45	0.040	0.017	0.096	3.2
*p* Cultivar**^‡^**	**0.0001**	**<0.0001**	**<0.0001**	**0.0010**	**<0.0001**	**<0.0001**	**<0.0001**	**<0.0001**
LSD **_(0.05)_**	0.165	0.0105	2.62	5.17	0.077	0.023	0.156	2.9

Each value is the mean of eight plants, one from each of the two watering treatments in each of the four sequential replications. Data are presented only for those traits that did not show a significant cultivar by water interaction effect.

**^‡^** Within a measured trait (column), significant cultivar main effects (*p* < 0.05) are indicated in bold.

Although the cultivars did not statistically differ for their WU under drought stress, *PRO 2715R* had the highest WU, 16.9 L plant^-1^, while *S08-C3* had the lowest WU, 14.7 L plant^-1^, which nominally amounted to a genetic WU variation of 15% (calculated as the [(maximum - minimum value)/minimum value **×** 100]). The cultivars did statistically differ for their WU under control watering treatment conditions; *Saska* had the highest WU, 37.0 L plant^-1^, while *OAC Lakeview* had the lowest WU, 28.5 L plant^-1^, which amounted to a genetic WU variation of 30% (**Table 3**).

Although there were no significant cultivar **×** water interaction effects for WUE and HI, under drought stress, *5A090RR2* had the highest WUE, 2.37 g L^-1^, while *Wildfire* had the lowest WUE, 1.83 g L^-1^, which amounted to a genetic variation in WUE of about 30%. Under control watering treatment conditions, *OAC Drayton* had the highest WUE, 2.08 g L^-1^, while *Saska* had the lowest WUE, 1.63 g L^-1^, which amounted to a genetic variation of 27%. Moreover, under drought stress, *OAC Lakeview* had the highest HI, 0.58 g g^-1^, while *PRO 2715R* had the lowest HI, 0.42 g g^-1^, which amounted to a genetic variation in HI of about 37%. Under control watering treatment conditions, *OAC Lakeview* had the highest HI, 0.54 g g^-1^, while *PRO 2715R* had the lowest HI, 0.41 g g^-1^, which amounted to a genetic variation in HI of about 33% (**Supplementary Table 1**).

Using cultivar means averaged across watering treatments, genetic variation of 21% was recorded for SPP, 51% for SW, 93% for RDM, 34% for TDM, 92% for R:S, 33% for HI, 24% for WUE, and 14% for DTM (**Table 4** **)**.

### Effects of cultivar and drought stress on soil water extraction

**Figure 3:**
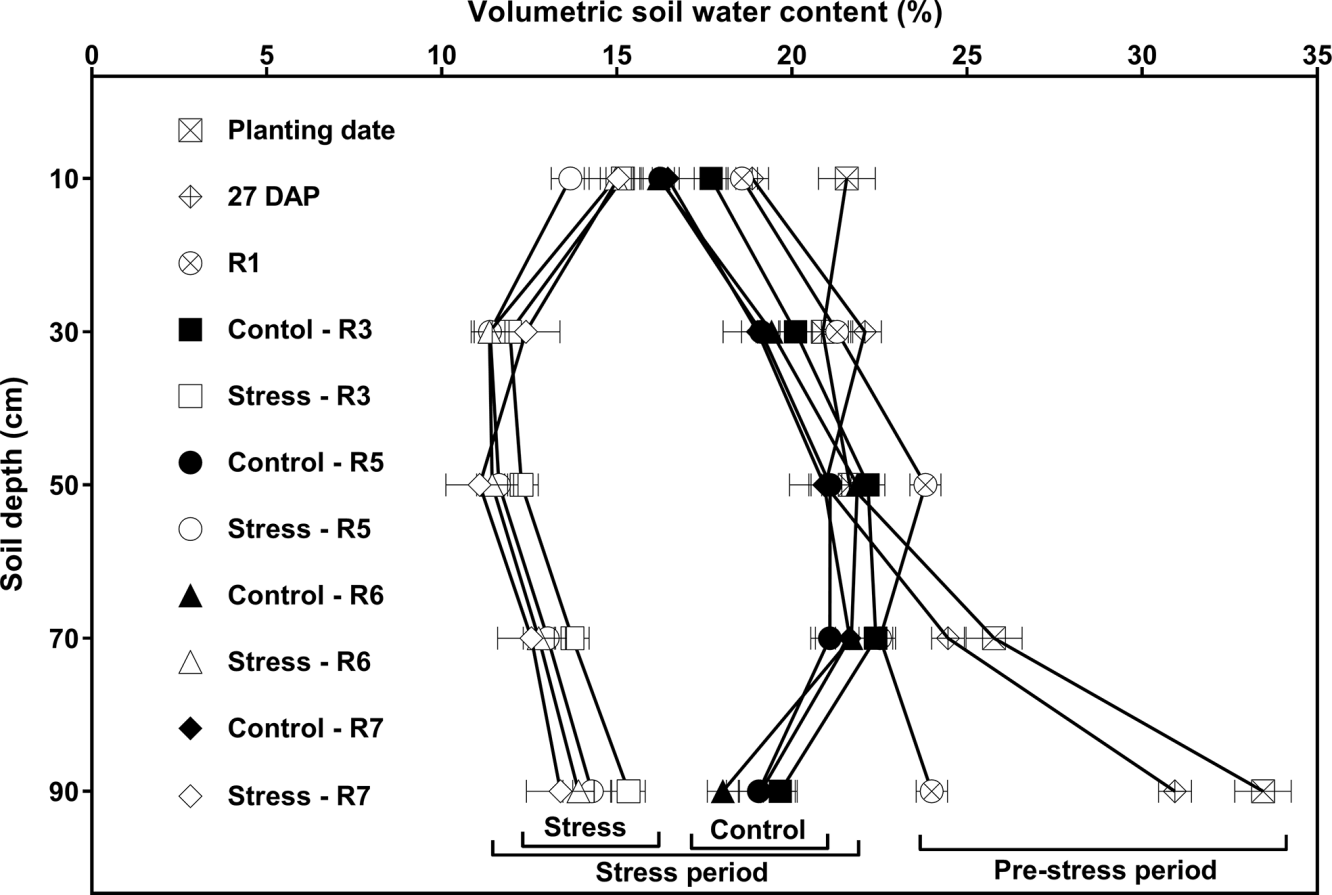
Effects of watering treatment and soil depth on volumetric soil water content (VSWC; %) at different developmental stages, averaged across 15 soybean cultivars and four replications. Watering treatments are watering daily to either 100% soil water holding capacity (SWHC; Control) or 50% SWHC (Stress). The stress treatment was imposed at the R1 developmental stage. The VSWC measurements were taken 24 h after the previous watering. Measurements were made during the pre-stress period [at planting date, 27 days after planting (DAP), and R1 stage; each value is the least squares mean of 120 data points] and stress period (at R3, R5, R6, and R7 stages; each value is the least squares mean of 60 data points). Error bars represent ± 1 S.E. and if not shown are smaller than the symbol.

**Figure 3** shows the effects of watering treatment and soil depth on VSWC across different developmental stages. Once the watering treatments were imposed (after the R1 stage), there were clear differences in VSWC profiles between the control and drought stress watering treatments throughout the remainder of the experiment, with the control watering treatment having a significantly higher VSWC at every depth as compared to the drought stress watering treatment.

Consistent with the effect of cultivar on WU shown in **Table 3**, the cultivar main effects on VSWC (averaged across five measurement depths and two watering treatments) were significant (*p *< 0.05) at several developmental stages, (R1, R5 and R7; **Supplementary Tables 2-5**), but no significant cultivar **×** water interaction effects were detected. Also, there were no significant cultivar **×** depth, or cultivar **×** water **×** depth interaction effects on VSWC. That is, there was no evidence that the cultivars differed in their ability to extract soil water from different depths in the soil profile at any developmental stage.

### Genetic variation for drought tolerance

**Table 5** shows the effects of cultivar on the drought stress to control ratios of SY (i.e., SYR), PN, SPP, SW, SDM, and WU. The cultivars differed significantly (*p* < 0.05) for the drought stress to control ratios of SY, PN, SW, and WU, but not for the drought stress to control ratios of SPP and SDM. Among the 15 cultivars, two drought-sensitive cultivars (*Saska* and *OAC Drayton*; SYR of ~ 0.44 each) and three drought-tolerant cultivars [*OAC Lakeview* (SYR of ~ 0.60), *OAC Champion* (SYR of ~ 0.54), and *PRO 2715R* (SYR of ~ 0.55)] were identified. That is, the drought-tolerant cultivars had a significantly higher SYR (lower fractional yield loss under drought stress) than the drought-sensitive cultivars (higher yield loss) (**Table 5**). Averaged across the 15 cultivars, the average drought stress to control ratios for SY, PN, SDM, and WU were each 0.49 (about 51% loss in SY, PN, SDM, and WU due to drought stress). In contrast, the average drought stress to control ratios for SPP and SW were 0.96 and 1.06, respectively (**Table 5**).

**Table 5:** Drought stress to control ratio for seed yield (SY; g g^-1^), pod number (PN; pods pods^-1^), seeds per pod (SPP; seeds seeds^-1^), single-seed weight (SW; g g^-1^), shoot dry matter (SDM; g g^-1^), and water use (WU; L L^-1^) for 15 soybean cultivars grown in a greenhouse under two watering treatments [control (100% soil water holding capacity; SWHC), and drought stress (50% SWHC) conditions] in 1-m rooting columns.

Ratio of drought stress to control plant						
Cultivar	SY	PN	SPP	SW	SDM	WU
5A090RR2	0.486	0.494	0.932	1.053	0.490	0.485
Absolute RR	0.472	0.465	0.965	1.059	0.466	0.487
Blade RR	0.474	0.427	0.992	1.101	0.493	0.499
Bruce	0.491	0.441	0.995	1.114	0.457	0.460
Dares	0.486	0.511	0.914	1.053	0.482	0.485
DH420	0.501	0.546	0.932	0.992	0.520	0.504
HDC 2701	0.461	0.418	0.963	1.143	0.439	0.494
OAC Champion	0.542**^+^**	0.505	0.986	1.087	0.547	0.537
OAC Drayton	0.443**^-^**	0.430	1.019	1.043	0.420	0.473
OAC Lakeview	0.601**^+^**	0.649	0.881	1.055	0.559	0.555
OAC Purdy	0.471	0.463	0.900	1.151	0.499	0.469
PRO 2715R	0.551**^+^**	0.494	0.996	1.134	0.521	0.502
S08-C3	0.471	0.555	0.871	0.978	0.489	0.480
Saska	0.437**^-^**	0.461	0.955	0.998	0.444	0.416
Wildfire	0.515	0.496	1.048	1.012	0.523	0.485
Mean	0.493	0.490	0.957	1.065	0.490	0.489
S.E.	0.052	0.053	0.051	0.039	0.050	0.033
CV (%)	11.8	16.5	10.2	7.4	12.2	8.8
*p* Cultivar**^‡^**	**0.0219**	**0.0238**	0.3839	**0.0431**	0.0714	**0.0267**
LSD **_(0.05)_**	0.083	0.115	0.139	0.113	0.085	0.062

**^+^** Cultivars marked with “**+**” have a significantly higher seed yield ratio (SYR) (*p* < 0.05) than cultivars marked with “-”, according to a protected Fisher’s LSD test. **^‡^** Significant cultivar effects (*p* < 0.05) are indicated in bold.

Data represent the least-squares mean values of four sequential replicates.

To test if drought tolerance (high SYR) was associated with a smaller effect of drought stress on days to maturity, we regressed SYR on the drought stress to control ratio for DTM. However, there was no relationship between the two drought stress to control ratios (r **=** 0.17; *p* ≥ 0.05; **Supplementary Figure 1**).

### Relationships between yield and other traits

**Figure 4:**
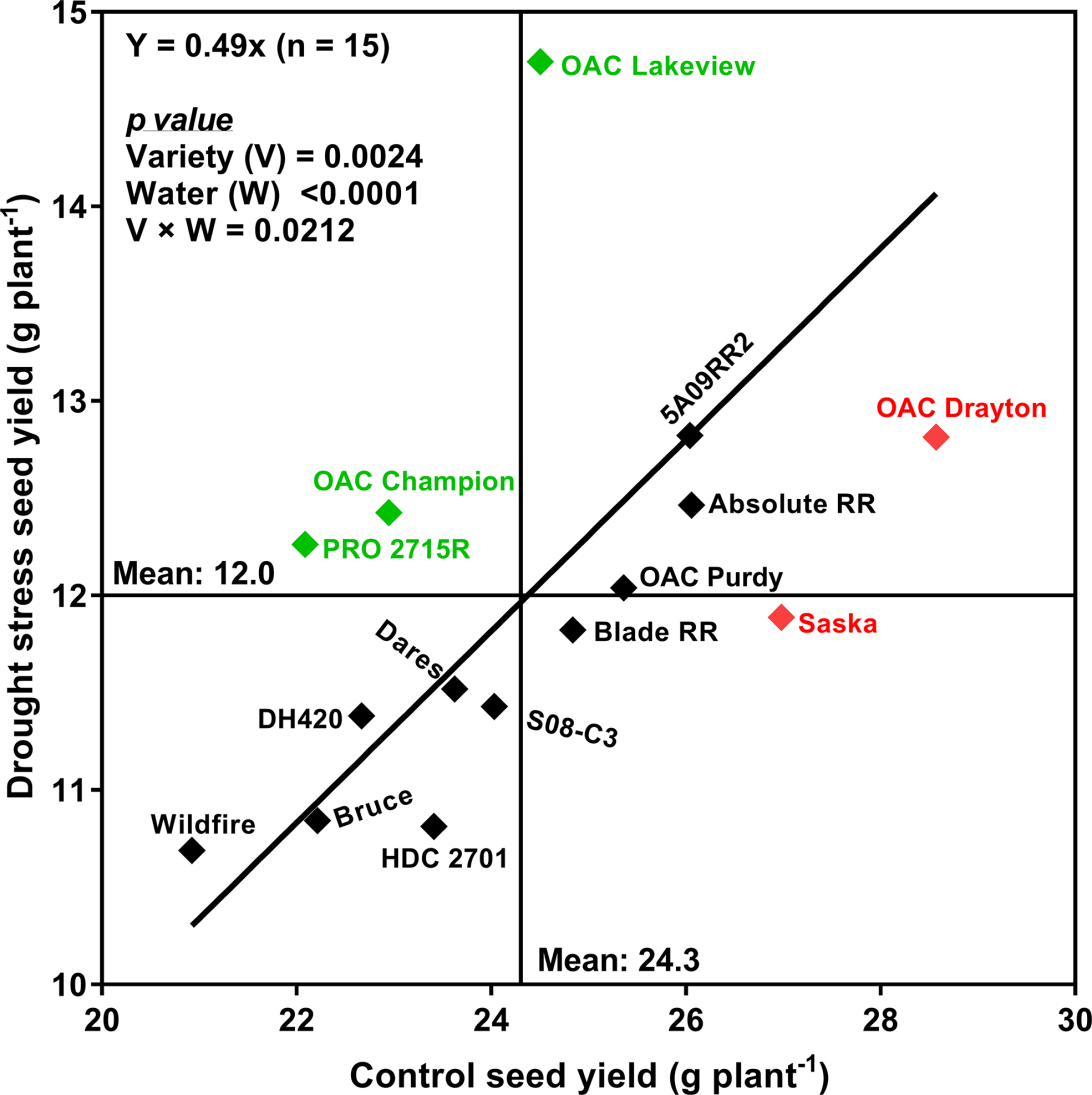
Relationship between seed yield under control (watered daily to 100% soil water holding capacity; SWHC) and drought stress (watered daily to 50% SWHC) conditions for the 15 soybean cultivars grown in a greenhouse in 1-m rooting columns in 2017 and 2018. The line is the best fit regression through the origin. Four sequential replicates were used.

**Figure 5:**
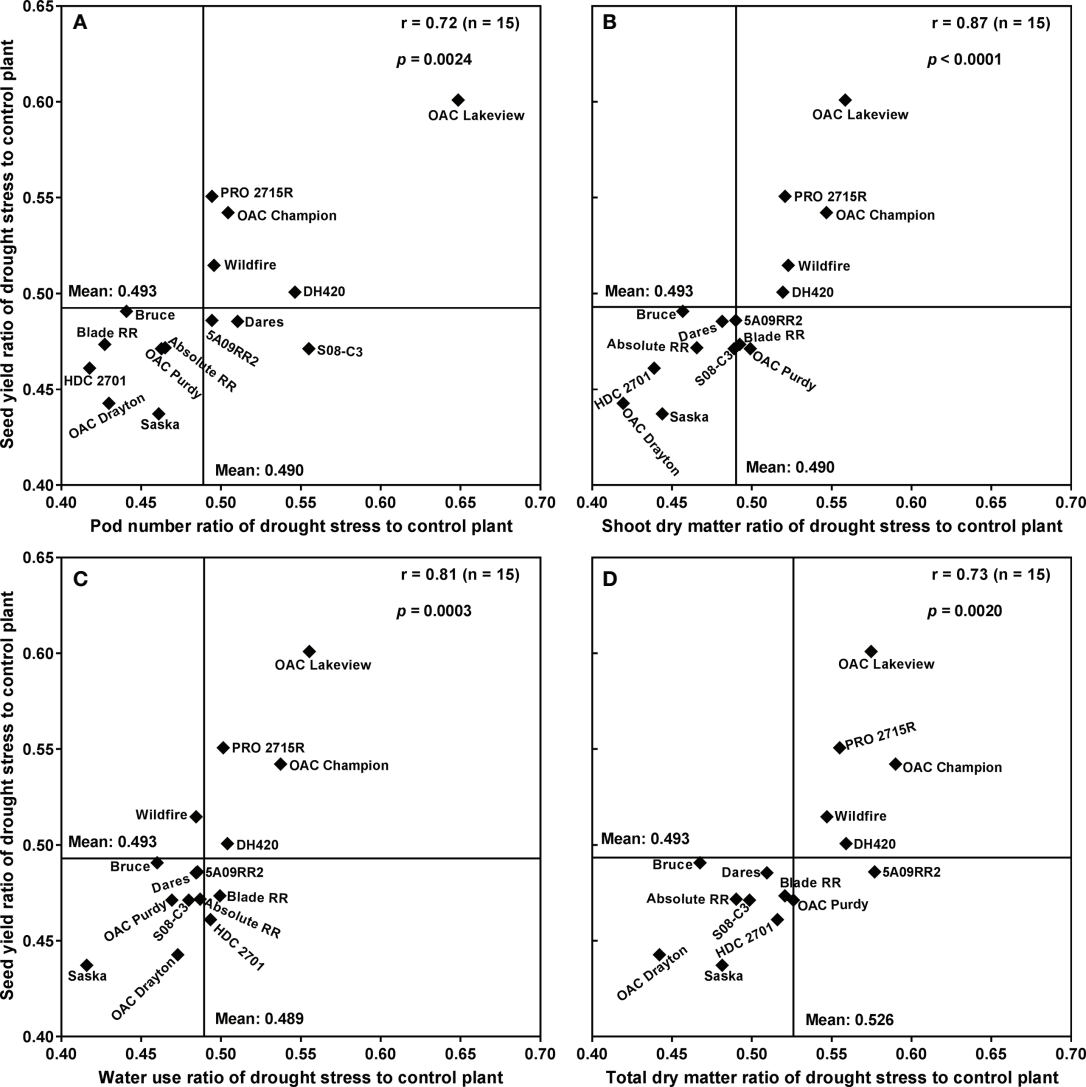
Relationships between the drought stress to control seed yield ratio and pod number ratio **(A)**, shoot dry matter ratio **(B)**, water use ratio **(C)**, and whole-plant dry matter ratio **(D)** for 15 soybean cultivars grown in a greenhouse in 1-m rooting columns in 2017 and 2018. Four sequential replicates were used.

**Figure 4** plots the mean SY under drought stress conditions against the mean SY under control conditions, for each cultivar, along with the best-fit regression line forced through the origin. Correlation analyses revealed that SYR was strongly associated with the drought:control ratios of PN (r **=** 0.72; *p* < 0.01), SDM (r **=** 0.87; *p* < 0.0001), WU (r **=** 0.81; *p* < 0.001) and TDM (r **=** 0.73; *p* < 0.01) (**Figure 5**). Moreover, there was a strong relationship between WU and TDM under drought stress (r **=** 0.68; *p* < 0.01); the drought stress to control ratio for WU also correlated with the ratio for TDM (r **=** 0.73; *p* < 0.01) (**Supplementary Table 6**) and the ratio for PN (r **=** 0.58; *p* < 0.05) (**Supplementary Table 6**).

High SDM-based WUE under drought stress was significantly associated with high SY under drought stress (r **=** 0.61; *p *< 0.05) and with high SYR (r **=** 0.54; *p *< 0.05) (**Supplementary Table 6**). However, under control conditions SDM-based WUE was not significantly associated with either SY (r **=** 0.29; *p* ≥ 0.05) or SYR (r = 0.15; *p* ≥ 0.05) (**Supplementary Table 6**). Furthermore, SDM under drought stress and the drought stress to control ratio of TDM were also predictive of SYR (r **=** 0.56; *p *< 0.05, and r **=** 0.73; *p *< 0.01, respectively) (**Supplementary Table 6**).

### PCA of drought stress to control ratios of yield and related traits

**Figure 6:**
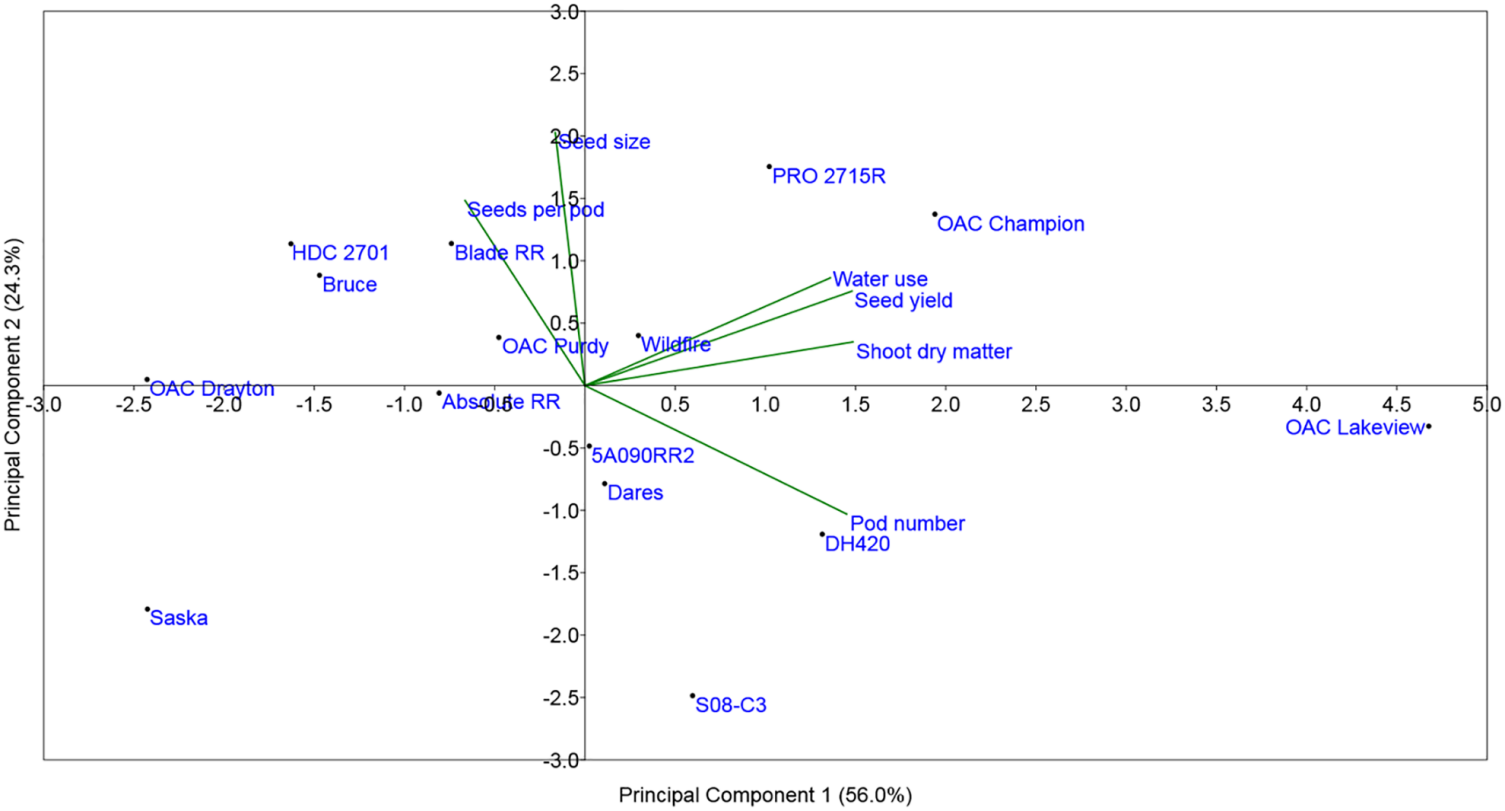
Principal Component Analysis (PCA) of seed yield, yield components, and related yield formation traits for the 15 commercial elite soybean cultivars adapted to Ontario grown in a greenhouse in 1-m rooting columns in 2017 and 2018. For all traits, the analysis was carried out using the ratio of the drought stress to the control ratio, determined from means across four replications.

**Figure 6** shows the biplot display of the Principal Component Analysis (PCA) of the drought stress to control ratios for SY, PN, SPP, SW, SDM, and WU for the 15 cultivars. The first principal component accounted for 56% of the total variation and was strongly influenced by the stress to control ratios of WU, SY, SDM, and to some extent, PN. The second principal component explained about 24% of the total variability and was strongly associated with the ratios for SW and to some extent SPP. The PCA analysis revealed that SYR was strongly associated with the drought stress to control ratios for WU, SDM, and to a lesser extent with the PN ratio. Drought stress to control ratios of the other two yield components, SW and SPP, appeared to be quite independent of SYR (i.e., their vectors on the PCA were at nearly 90 degrees to the SYR vector). The PN vector was approximately opposite to the vectors for the other two yield components (SW and SPP), indicating that cultivars that had largest reductions in PN under drought stress tended to compensate with higher values of SW and/or SPP. As expected, the drought-tolerant cultivars (*OAC Lakeview*, *OAC Champion*, and *PRO 2715R*) had the longest SYR vectors, whereas the drought-sensitive cultivars (*Saska* and *OAC Drayton*) had the shortest ones, which were clustered on the opposite side to the drought-tolerant cultivars. Overall, the PCA indicates that the drought-tolerant cultivars were generally those that maintained high WU and SDM and, to a lesser extent, high PN, under drought stress conditions relative to control watering treatment conditions (**Figure 6**; **Table 5**). In contrast, the drought-sensitive cultivars were those that had lower drought stress to control ratio values for these traits (**Figure 6**; **Table 5**).

## Discussion

The main purpose of this study was to test the hypothesis that commercial soybean cultivars adapted to Ontario differ in their tolerance to drought stress (defined as the ratio of their seed yield under drought conditions compared to control conditions; SYR) and to characterize the physiological basis for any such differences in drought tolerance. Specifically, we wanted to see how a high SYR was related to drought effects on yield components (PN, SPP, and SW), plant WU and dry matter (DM) production.

While not producing symptoms of severe stress such as leaf wilting, which are also not common under Ontario field conditions, the drought stress treatment that was imposed through the entire reproductive period (R1 to R8 developmental stages) resulted in a significant reduction in SY as well as every other trait that we measured, except for SW and HI. When averaged across the cultivars, the drought stress treatment reduced final plant DM and total plant WU by about 50%. SY was also reduced by about 50%, and almost all of this reduction could be attributed to a single yield component – PN. SPP decreased significantly under drought stress, and SW increased significantly, but these changes were very small compared to the change in PN. Effects of drought on DTM were also statistically significant, but small. These findings are consistent with our previous study and further indicate that PN is the most important yield component driving SY response to drought stress in this culture system (Gebre, 2020; Gebre and Earl, 2021), which is also typically observed in the field. Other studies have also reported similar effects of drought stress on soybean SY (He et al., 2016; He et al., 2017; Giordani et al., 2019; Gebre and Earl, 2021), WU (Earl, 2002; He et al., 2016; He et al., 2017; Gebre and Earl, 2020; Gebre and Earl, 2021), TDM (Earl, 2002; He et al., 2016; Gebre and Earl, 2020; Gebre and Earl, 2021), PN (Snyder et al., 1982; He et al., 2017; Gebre and Earl, 2021), and DTM (Earl, 2012; Visser, 2014).

As expected, the drought stress treatment increased R:S and WUE. This indicates a greater proportion of DM allocation to the roots and a more efficient utilization of available water (i.e., more DM produced per unit of water used) under drought stress conditions. Similar increases in R:S and WUE under drought stress have been reported previously (e.g., Earl, 2002; Gebre and Earl, 2020; Gebre and Earl, 2021). In contrast, HI was not responsive to watering treatment as about 50% of the total SDM was allocated to the seed in both watering treatments. Contrary to our results, reduced HI under drought stress has been reported in soybean in some past research (e.g., Giordani et al., 2019; Oya et al., 2004). However, a significantly higher HI was reported in a single Ontario-adapted commercial soybean cultivar *OAC Bayfield* grown under drought stress (about 16% higher than the control) in a previous study (Gebre and Earl, 2021), while He et al. (2016) reported no significant difference in HI between drought stress and control watering treatments for two old and two new soybean cultivars.

Overall, this experiment supports the following model of yield reduction due to drought stress: the drought stress treatment reduces whole-plant WU, which produces a nearly proportional decrease in whole-plant DM (since WUE increased only slightly) and in SY, with PN being the yield component most affected. This is consistent with the more general model that PN and thereby SN, primarily determines SY in soybean, with PN being strongly determined by plant growth rates during the “critical period” from about R2 to R5.5 (e.g., Vega et al., 2001). Also consistent with this model, cultivars that were able to use water to produce more yield did so by producing more TDM and more specifically SDM, not by allocating a larger fraction of that SDM to the seed.

There were significant cultivar **×** watering treatment interaction effects for SY, PN, SDM, and WU, indicating that the cultivars differed for their response to drought stress for those traits. The significant cultivar **×** treatment interaction effect for SY suggests that there is genetic variation for drought tolerance among these 15 cultivars. Similar to SY, the cultivars also differed for PN loss and SDM reduction under drought stress conditions. All four traits that showed significant cultivar **×** water interaction effects also showed a very uniform average 51% reduction due to drought stress, suggesting a quantitative, mechanistic relationship between them. Similar to our findings, He et al. (2017; 2019) reported significant genotype **×** treatment interaction effects in soybean for SY, PN, SDM, and WU, while Giordani et al. (2019) reported significant genotype **×** treatment interaction effects for SY and PN but not for SDM (they did not measure WU in their work). Fried et al. (2019) also reported a significant genotype **×** watering treatment interaction effect for SY among 10 soybean genotypes at one of their experimental locations.

Similar genetic variation in susceptibility to yield loss under typical rainfed conditions in Ontario has been reported by Visser (2014). The author found that in a three-year field study, cultivars that increased their SDM the most under irrigation also showed the largest yield response to irrigation, while HI was quite stable across years, treatments, and cultivars. This finding is also in agreement with our results where HI was unaffected by drought stress, although in our experiment the cultivar main effect for HI was significant, indicating genetic variation for this trait.

Based on their SYR, two drought-sensitive cultivars (*Saska* and *OAC Drayton*) and three drought-tolerant cultivars (*OAC Lakeview*, *OAC Champion*, and *PRO 2715R*) were identified. We chose SYR *a priori* as an index of drought tolerance, but it must be recognized that SYR is increased when either the SY under drought stress is higher than expected, or the SY under control conditions is lower than expected. The latter case raises the possibility that cultivars identified as drought-sensitive according to SYR may just actually be unusually productive when water is plentiful. Indeed, both cultivars identified as being particularly drought-sensitive (*OAC Drayton* and *Saska*) had much higher than average yields under control conditions, while all three “drought-tolerant” cultivars had average (*OAC Lakeview*) or below average (*OAC Champion* and *PRO 2715R*) yields under control conditions. This may indicate that cultivars with high SYR are systematically those with poor yields under well-watered conditions, or it may just be a coincidence, unique to this particular set of cultivars. The latter possibility is unlikely given that cultivars such as *OAC Lakeview* and *OAC Champion* have been grown by Ontario farmers for close to two decades and still perform reasonably well for yield as compared to the more recent, modern ones. However, a broader selection of germplasm should be evaluated to determine if in fact high SYR is generally associated with average or low yield potential. Even if that is the case, a cultivar such as *OAC Lakeview* would still warrant further investigation, given that i) it produced the highest yield under drought stress while still producing average yield in the absence of drought stress, and ii) its mode of drought tolerance appeared to be unique, with PN being unusually insensitive to drought stress (stress:control PN ratio of 0.649, by far the highest of any of the cultivars tested (**Table 5**).

In the present study, SYR was positively and strongly associated with the stress:control ratios of both TDM and WU, consistent with our previous findings with soybean in this culture system that WU and TDM are closely tied (Gebre, 2020; Gebre and Earl, 2020; Gebre and Earl, 2021). This raises the prospect that soybean drought tolerance could be usefully assayed in real time and non-destructively by measuring WU alone. In other words, if biomass accumulation during some critical developmental period strongly predicts SY, and WU predicts biomass accumulation, then a non-destructive measure of the response of WU to stress during that period should be a useful measure of drought tolerance.

The PCA of the drought stress to control ratios for SY, PN, SPP, SW, SDM, and WU for the 15 cultivars further supported most of the above relationships. It confirmed that SYR was strongly associated with the drought stress to control ratios of WU, SDM, and to a lesser extent with PN ratio, further supporting the idea that increasing WU under drought stress significantly increased SY by increasing SDM and PN. In general, the drought-tolerant cultivars were those that continued to use water under drought stress conditions to produce a significantly higher DM accumulation and higher PN compared to the drought-sensitive cultivars. By contrast, the cultivars that slowed their WU (i.e., shut down to an extent) under drought stress suffered more in terms of the loss to SDM and SY.

The PCA also revealed the expected relationships between changes in the various yield components. Among them, PN was the primary driver and predictor of SY, as compared to SW and SPP, but there was also evidence of the expected compensation between yield components. For example, those cultivars that saw the largest reductions in PN under stress (lower PN ratio) tended to be those that had higher stress:control ratios for SW and especially SPP, as also observed by Visser (2014) in field trials. These slightly different modes of drought tolerance among cultivars are evident in the PCA, where for example, *OAC Champion* and *PRO 2715R* are more closely associated with the SW ratio vector, and *OAC Lakeview* with the PN ratio vector.

In the current study, the lack of significant cultivar **×** watering treatment interaction effect for DTM and the lack of association between SYR and the stress:control ratio for DTM indicate that cultivar differences in SY response to drought stress cannot be attributed to differences in the way drought stress affected maturity (i.e., by differentially shortening the seed-filling period).

The total genetic variation for WUE in this experiment was 24%. Hufstetler et al. (2007) also reported similar total genetic variation (~25%) for WUE among 23 soybean cultivars, breeding lines and plant introductions. We also found a significant positive association between WUE under stress (only) and SYR. In interpreting this result, it should be noted that WUE under stress is mathematically auto-correlated with the stress:control ratio for SDM (which itself is positively correlated with SYR); however, it is *negatively* auto-correlated with the stress:control ratio for WU, (which is also positively correlated with SYR). Hence, on balance, it appears that the correlation between WUE under stress and SYR is not simply a mathematical artefact. High WUE can arise from reduced transpiration (e.g., through reduced stomatal conductance, leading to soil water conservation) or from higher photosynthetic response to leaf internal CO_2_ concentration (Earl, 2002). The design of the present experiment would have disadvantaged plants with the former strategy, since all transpired water was replaced on a daily basis regardless of the daily WU. This suggests that in this experiment it was the higher photosynthetic response to leaf internal CO_2_ under drought stress that gave high-WUE cultivars a yield advantage. It is not clear how this would translate to a field environment where soil water conservation might also be advantageous.

In conclusion, drought stress significantly affected every trait we measured, except HI. There exists substantial genetic variation among Ontario-adapted commercial soybean cultivars for their response to drought stress, and there was a broad range of genetic variation for SYR among the cultivars tested. Based on their SYR, we identified two drought-sensitive cultivars (*Saska* and *OAC Drayton*) and three drought-tolerant cultivars (*OAC Lakeview*, *OAC Champion*, and *PRO 2715R*). Drought-tolerant cultivars were those that maintained higher WU, SDM, and PN under drought stress conditions. However, the different cultivars had slightly different modes of drought tolerance in terms of yield components (PN, SPP, or SW). Our study helps to define the physiological basis of soybean cultivar differences in drought tolerance, and provides direction for soybean breeders wishing to target specific traits that could improve soybean yield when soil water is limiting.

## Data availability statement

The original contributions presented in the study are included in the article/**Supplementary Material**. Further inquiries can be directed to the corresponding author.

## Author contributions

MG performed the experiment, data collection, statistical data analysis and presentation, and drafted the manuscript. MG and HE conceived the project and experimental design and collaborated on the data interpretation and manuscript revision. IR assisted with cultivar selection, project management and manuscript revision. All authors contributed to the article and approved the submitted version.

## Funding

This research was funded by Grain Farmers of Ontario (Project number: GF2 0122) and the Natural Sciences and Engineering Research Council of Canada (Grant number: CRDPJ 513541 – 17).

## Acknowledgments

Financial support from the Grain Farmers of Ontario (Project number: GF2 0122) and the Natural Sciences and Engineering Research Council of Canada (Grant number: CRDPJ 513541 – 17) is gratefully acknowledged. The authors are also grateful to Godfrey Chu (University of Guelph), Laxhman Ramsahoi (University of Guelph) and Austin Bruch (University of Guelph) for their technical assistance.

## Conflict of interest

The authors declare that the research was conducted in the absence of any commercial or financial relationships that could be construed as a potential conflict of interest.

## Publisher’s note

All claims expressed in this article are solely those of the authors and do not necessarily represent those of their affiliated organizations, or those of the publisher, the editors and the reviewers. Any product that may be evaluated in this article, or claim that may be made by its manufacturer, is not guaranteed or endorsed by the publisher.

## Abbreviations

CV, coefficient of variation (%)**;** DTM, days to maturity**;** HI, harvest index**;** PN, pod number (pods plant^-1^); SN, seed number (seeds plant^-1^)**;** RDM, root dry matter (g plant^-1^); R:S, root to shoot dry matter ratio**;** SDM, shoot dry matter (g plant^-1^)**;** SPP, seeds per pod**;** SW, single-seed weight (g seed^-1^); SWHC, soil water holding capacity; SY, seed yield (g plant^-1^)**;** SYR, seed yield ratio (ratio of yield under drought conditions to yield under control conditions); TDM, total dry matter (g plant^-1^); TDR, time-domain reflectometry; VSWC, volumetric soil water content (%); WU, water use (L plant^-1^); WUE, water use efficiency (g L^-1^).
