# Supplementary material for: Genetic variation for effects of drought stress on yield formation traits among commercial soybean [Glycine max (L.) Merr.] cultivars adapted to Ontario, Canada
Source: Front Plant Sci. 2022 Oct 13;13:1020944. doi: 10.3389/fpls.2022.1020944 (PMC9612836; doi:10.3389/fpls.2022.1020944)
Supplement: Supplementary file 1 [file DataSheet_1.pdf]

Supplementary Material

Supplementary Table 1: Cultivar and watering treatment interactive effects on harvest index (HI; g g^{-1}) and whole-plant dry matter-based water use efficiency (WUE; g L^{-1}) for 15 soybean cultivars grown in a greenhouse in 1-m rooting columns under two watering treatments [control (100% soil water holding capacity; SWHC), and drought stress (50% SWHC)]. Each value is the mean of four plants, one from each of the four sequential replications.

Yield and related traits	Harvest index (g g^{-1})		Water use efficiency (g L^{-1})	
Cultivar	Stress	Control	Stress	Control
5A090RR2	0.53	0.53	2.37	2.00
Absolute RR	0.53	0.53	2.05	2.03
Blade RR	0.51	0.53	2.10	2.06
Bruce	0.46	0.43	2.05	2.03
Dares	0.47	0.46	2.07	1.98
DH420	0.51	0.54	1.89	1.71
HDC 2701	0.52	0.49	1.98	1.92
OAC Champion	0.50	0.51	2.20	2.00
OAC Drayton	0.54	0.50	1.94	2.08
OAC Lakeview	0.58	0.54	2.05	1.98
OAC Purdy	0.49	0.52	1.98	1.79
PRO 2715R	0.42	0.41	2.30	2.07
S08-C3	0.52	0.54	1.91	1.86
Saska	0.52	0.53	1.90	1.63
Wildfire	0.49	0.50	1.83	1.64
p Cultivar‡	**<0.0001**		**<0.0001**	

‡Within a measured trait, significant cultivar main effects ($p < 0.05$) are indicated in bold.

Supplementary Table 2: A generalized linear mixed model repeated measures analysis of the effects of cultivar, watering treatment, and soil depth on volumetric soil water content (VSWC; %) for 15 commercial soybean varieties adapted to Ontario grown in a greenhouse in 1-m rooting columns in 2017 and 2018. Drought stress treatments are watering daily to either 100% soil water holding capacity (SWHC; control) or 50% SWHC (drought stress). Drought stress was imposed at the R1 developmental stage. The VSWC measurements were taken 24 h after the previous watering. The measurements were made during the pre-stress period [at 27 days after planting (DAP), and R1 stage] and stress period (at R3, R5, R6, and R7 stages). Data represent the cultivar least-square means across the five profile depths during the pre-stress period, and the cultivar least-square means across the two watering treatments and five profile depths during the stress period. Four sequential replicates were used.

	**Pre-stress period**		**Stress period**			
Cultivar	27 DAP	R1	R3	R5	R6	R7
5A090RR2	23.6	22.6	17.1	16.0	16.2	15.4
Absolute	23.5	22.4	17.2	16.3	16.4	15.7
Blade RR	23.6	22.4	17.1	16.3	15.8	15.4
Bruce	23.4	22.6	17.1	15.6	15.9	15.8
Dares	23.7	22.6	17.3	16.4	16.5	16.2
DH420	23.0	21.7	16.7	15.7	16.1	17.0
HDC2701	23.4	21.7	17.4	16.5	16.5	17.1
OAC Champion	23.3	22.2	17.0	16.1	16.3	16.5
OAC Drayton	23.5	21.5	16.8	15.8	16.1	16.0
OAC Lakeview	23.6	21.6	17.2	16.3	16.1	16.4
OAC Purdy	23.4	21.3	16.7	15.7	15.7	16.8
PRO2715R	23.6	21.9	17.1	15.8	16.1	15.2
S08C3	23.7	22.3	17.3	16.5	16.5	16.8
Saska	23.6	21.7	16.7	15.6	15.9	15.9
Wildfire	23.1	22.1	16.9	16.3	16.5	16.3
Mean	23.5	22.0	17.0	16.1	16.2	16.2
p Cultivar‡	0.0847	**0.0006**	0.3728	**0.0156**	0.2491	**0.0159**
LSD **(0.05)**	0.67	0.98	0.82	0.67	0.73	0.87

‡Within a column, significant variety main effects ($p < 0.05$) are indicated in bold. A least significant difference (LSD) among the varieties was estimated according to a protected Fisher's LSD test.

Supplementary Table 3: A generalized linear mixed model repeated measures analysis of the effects of variety and soil depth on volumetric soil water content (VSWC; %) for 15 soybean varieties grown in a greenhouse in 1-m rooting columns under control watering treatment (100% soil water holding capacity) conditions. The VSWC measurements were taken 24 h after the previous watering at the R1 developmental stage, prior to the initiation of the drought stress treatments. Data represent the mean values of four sequential replicates.

Random effects	Subject	Estimate	Standard error	ChiSq	Pr > ChiSq‡
Block (B)		0.6374	0.5343	65.41	**<0.0001**
CS	B × Variety	-0.5733	0.1035		
Residual		5.4215	0.3741		
Fixed effects	Num df	Den df	F value	Pr > F‡	
Variety (V)	14	102	3.05	**0.0006**	
Depth (D)	4	420	109.54	**<0.0001**	
V × D	56	420	0.78	0.8726	

‡Significant effects ($p < 0.05$) are indicated in bold.

Supplementary Table 4: A generalized linear mixed model repeated measures analysis of the effects of variety, watering treatment, and soil depth on volumetric soil water content (VSWC; %) for 15 soybean varieties adapted to Ontario grown in a greenhouse in 1-m rooting columns. Drought stress treatments are watering daily to either 100% soil water holding capacity (SWHC; control) or 50% SWHC (drought stress). Drought stress was imposed at the R1 developmental stage. The VSWC measurements were taken 24 h after the previous watering at the R5 developmental stage. Four sequential replicates were used.

Random effects	Subject	Estimate	Standard error	ChiSq	Pr > ChiSq‡
Block (B)		1.0024	0.827	78.14	**<0.0001**
B × V		-0.2313	0.06064	11.53	**0.0007**
AR(1)	B × V × W	0.2775	0.05395		
Residual		2.4773	0.1967		

Fixed effects	Num df	Den df	F value	Pr > F‡
Variety (V)	14	14.5	3.28	**0.0156**
Water (W)	1	7.092	1013.38	**<0.0001**
V × W	14	5.524	0.45	0.8955
Depth (D)	4	61.14	33.99	**<0.0001**
V × D	56	48.99	0.77	0.8249
W × D	4	61.14	102.1	<**0.0001**
V × W × D	56	48.99	0.53	0.9880

‡Significant effects ($p < 0.05$) are indicated in bold.

Supplementary Table 5: A generalized linear mixed model repeated measures analysis of the effects of variety, watering treatment, and soil depth on volumetric soil water content (VSWC; %) for 15 soybean varieties adapted to Ontario grown in a greenhouse in 1-m rooting columns. Drought stress treatments are watering daily to either 100% soil water holding capacity (SWHC; control) or 50% SWHC (drought stress). Drought stress was imposed at the R1 developmental stage. The VSWC measurements were taken 24 h after the previous watering at the R7 developmental stage. Four sequential replicates were used.

Random effects	Subject	Estimate	Standard error	ChiSq	Pr > ChiSq‡
Block (B)		3.5277	2.8939	106.2	**<0.0001**
B × V		-0.03949	0.08169	3.03	0.0817
CS	B × V × W	-0.2061	0.1351		
Residual		3.9197	0.2922		

Fixed effects	Num df	Den df	F value	Pr > F‡
Variety (V)	14	6.278	6.04	**0.0159**
Water (W)	1	21.2	823.66	**<0.0001**
V × W	14	12.76	0.87	0.6031
Depth (D)	4	23.43	9.97	**<0.0001**
V × D	56	23.17	0.74	0.8239
W × D	4	23.43	85.89	**<0.0001**
V × W × D	56	23.17	0.76	0.7989

‡Significant effects ($p < 0.05$) are indicated in bold.

Supplementary Table 6: Relationships between seed yield and yield formation traits under drought stress and control conditions, and their drought stress to control ratio values for 15 soybean cultivars grown in a greenhouse in 1-m rooting columns in 2017 and 2018. The traits include: pod number ratio (PNR; g g^{-1}), seed yield under stress (SYS; g $plant^{-1}$), seed yield under control (SYC; g $plant^{-1}$), seed yield ratio (SYR; g g^{-1}), shoot dry matter under stress (SDMS; g $plant^{-1}$), total dry matter under stress (TDMS; g $plant^{-1}$), total dry matter ratio (TDMR; g g^{-1}), water use under stress (WUS; L $plant^{-1}$), water use ratio (WUR; L L^{-1}), shoot dry matter-based water use efficiency under stress (SWUES; g L^{-1}), and shoot dry matter-based water use efficiency under control (SWUEC; g L^{-1}) conditions. Four sequential replicates were used (n = 15).

Trait	SYS	SYC	SYR	SDMS	TDMS	TDMR	WUS	WUR	SWUES	SWUEC
**PNR**	0.52*	-0.23 ns	0.72 ns	0.14 ns	-0.08 ns	0.55 ns	-0.05 ns	0.58*	0.25 ns	-0.05 ns
**SYS**		0.48 ns	0.49 ns	0.57*	0.42 ns	0.36 ns	0.21 ns	0.49 ns	0.61*	0.47 ns
**SYC**			-0.53*	0.11 ns	0.05 ns	-0.39 ns	-0.14 ns	-0.34 ns	0.18 ns	0.29 ns
**SYR**				0.56*	0.34 ns	0.73*	0.37 ns	0.80***	0.54*	0.15 ns
**SDMS**					0.80***	0.23 ns	0.67**	0.27 ns	0.91****	0.64**
**TDMS**						0.44 ns	0.68**	0.26 ns	0.60**	0.49 ns
**TDMR**							0.25 ns	0.73**	0.28 ns	-0.08 ns
**WUS**								0.03 ns	0.32 ns	0.02 ns
**WUR**									0.32 ns	0.36 ns
**SWUES**										0.77***

* = $p < 0.05$, ** = $p < 0.01$, *** = $p < 0.001$, **** = $p < 0.0001$, ns = not significant ($p \geq 0.05$).

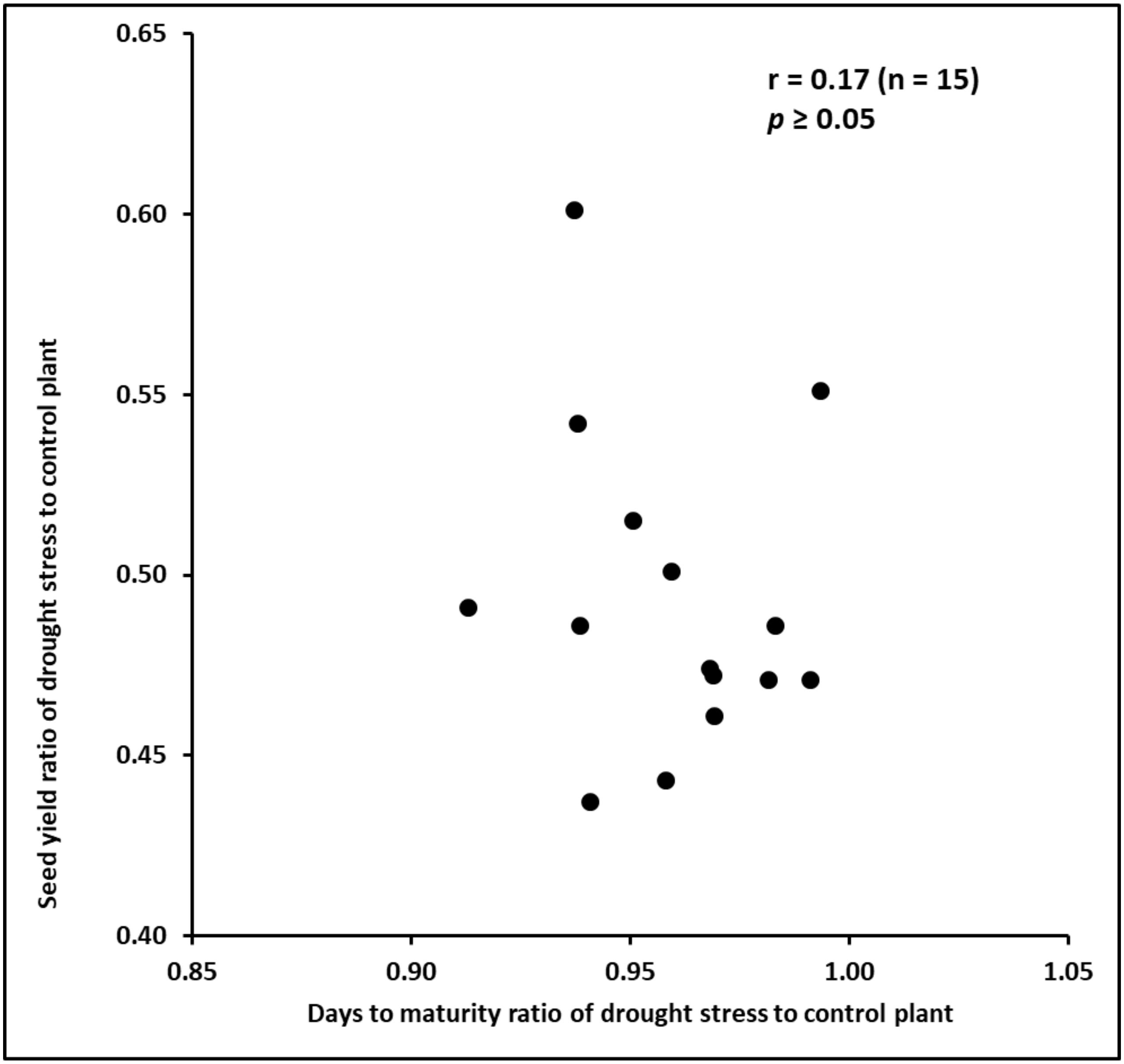

Supplementary Figure 1: Relationship between the drought stress to control seed yield ratio and days to maturity ratio for 15 soybean cultivars grown in a greenhouse in 1-m rooting columns in 2017 and 2018. Four sequential replicates were used.
